# Improved fixation stability for repairing pedicle screw loosening using a modified cement filling technique in porcine vertebrae

**DOI:** 10.1038/s41598-022-06724-4

**Published:** 2022-02-17

**Authors:** Ming-Kai Hsieh, Yun-Da Li, Yu-Chen Li, Mu-Yi Liu, Tsung-Ting Tsai, Po-Liang Lai, Ching-Lung Tai

**Affiliations:** 1 Department of Orthopaedic Surgery, Spine Section, Bone and Joint Research Center, Chang Gung Memorial Hospital, Chang Gung University College of Medicine, Taoyuan, Taiwan; 2 Graduate Institute of Biomedical Engineering, Chang Gung University, 33302 Taoyuan, Taiwan; 3 Ph.D. Program in Biomedical Engineering, Collage of Engineering, Chang Gung University, Taoyuan, Taiwan

**Keywords:** Biomedical engineering, Orthopaedics

## Abstract

Polymethylmethacrylate (PMMA) has been applied clinically and biomechanically repair loose pedicle screws. Controversies have arisen over data due to uncontrolled cement properties, various locations and sizes of fenestrated holes in repair screws, irregular holes and different bone densities of specimens. In this study, the pullout strength was compared for two techniques, the modified technique to use PMMA to augment a threaded hole and the traditional technique with retrograde injection of a PMMA filling, for standard loose screws in porcine vertebrae. Both techniques provided statistically significant results for sufficiently randomized specimens and experimental procedures. The difference in the pullout strength between conical and cylindrical screws for the aforementioned cement augmentation techniques was also investigated. Twenty-four single-level fresh-frozen lumbar vertebrae from L1 to L6 were harvested from four mature pigs. A total of 0.8 ml of PMMA was retrograde injected into screw holes with a 5.5 mm diameter, followed by insertion of a 5.0 mm diameter repair screw in the traditional group (n = 12). A stiff threaded PMMA hole was created with a 4.5 mm tapping screw before insertion of repair screws in the modified group (n = 12). Two screw geometries were randomly assigned as cylindrical (n = 6) and conical (n = 6) in each group. The correlations between filling techniques, screw geometries and axial pullout strength were analyzed. An appropriate screw trajectory and insertion depth were confirmed using X-ray imaging prior to pullout testing in both groups. For a given screw geometry (cylindrical or conical), the pullout force of the modified group was significantly higher than that of the traditional group. There was no significant difference in the pullout force between the screw geometries for a given filling technique. The cement augmentation technique is far more influential than the screw outer geometry. The modified PMMA technique created a greater anchor force than the traditional method and could be an alternative for revision of pedicle screw loosening.

## Introduction

Pedicle screw fixation is a common and useful procedure for treating various spinal disorders, including fracture, deformity and instability. Pedicle screw loosening is one of most frequently reported complications associated with the necessity of revision surgery, with a reported rate ranging from 0.8 to 50% and possibly, even higher in patients with osteoporosis^1–3^. Increased screw size and augmentation of loose screws with polymethylmethacrylate (PMMA) cement are commonly used techniques in revision surgery^4,5^. However, screws with PMMA-augmented cement are preferable because screw stability cannot be achieved only by increasing the screw diameter in a halo screw hole^6–10^.

Various PMMA augmentation techniques have been used in previous studies, including direct injection of PMMA into the hole of the vertebral body prior to screw insertion^8,11,12^. However, cement leakage along the broken pedicle hole can cause devastating results^11,12^. Other studies have included the use of perforated cannulated screws, which improved the screw anchoring strength by allowing the injection of cement through the perforation^13^. A study showed that solid screws with retrograde cement prefilling exhibited notably higher pullout strength than cannulated screws with cement injection through perforation^14^. Both perforated cannulated screws and solid screws with cement filling techniques had better pullout performance than screws without cement augmentation.

A modified transpedicular screw augmentation method was compared to the traditional filling method and used to repair loose pedicle screws^11,15^; however, the results were unreliable because there were too few specimens, the specimens had mixed bone density, irregular loosening holes were formed and the traditional filling method was not explained.

In this study, the pullout strength was compared for two techniques, the modified technique to use PMMA to augment a threaded hole and the traditional technique with retrograde injection of a PMMA filling, for standard loose screws in porcine vertebrae. Both techniques provided statistically significant results for sufficiently randomized specimens and experimental procedures. The difference in pullout strength between conical and cylindrical screws based on the aforementioned cement augmentation techniques was also investigated.

## Results

### Image analysis

**Figure 1:**
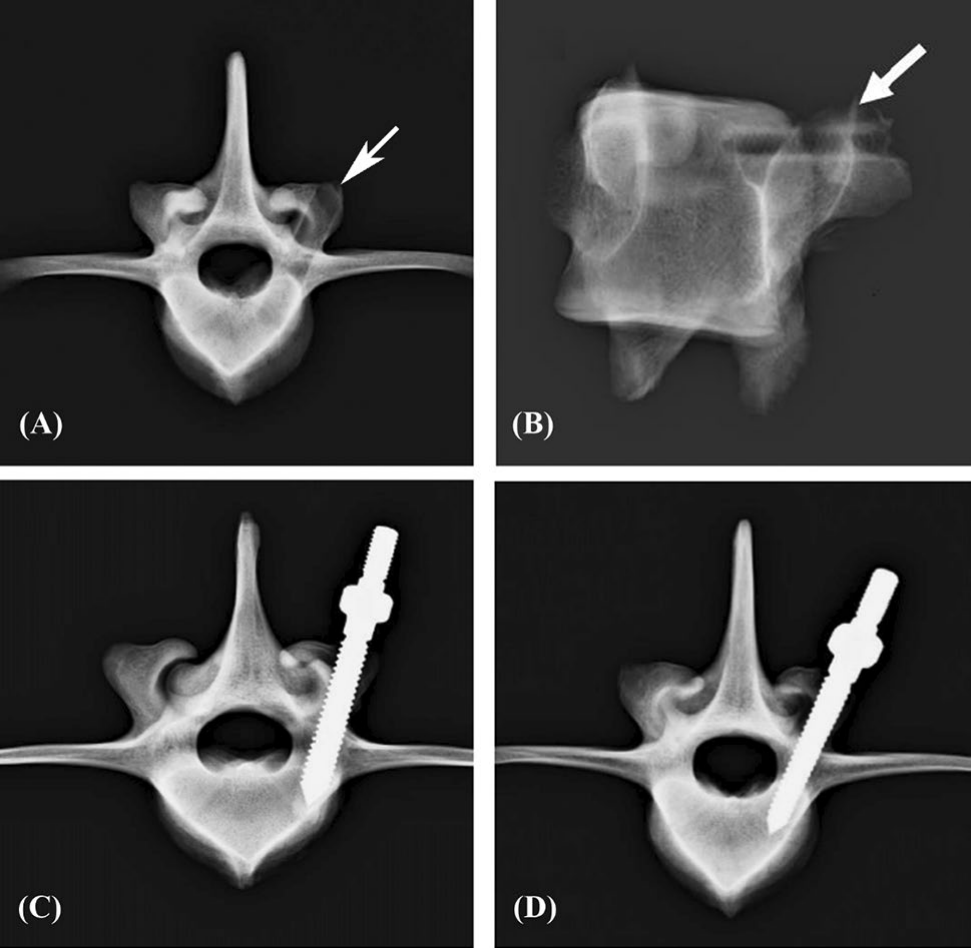
X-ray images of the traditional group. An enlarged screw hole was created and confirmed using axial (**A**) and sagittal X-ray (**B**) images prior to implantation (white arrow). A cylindrical (**C**) or conical (**D**) repair screw was convergently inserted into the 5.5 mm diameter enlarged screw hole after it was filled with PMMA. The tip of the inserted screw did not exceed the enlarged depth, and no signs of fracture or damage were detected in the vertebrae.

**Figure 2:**
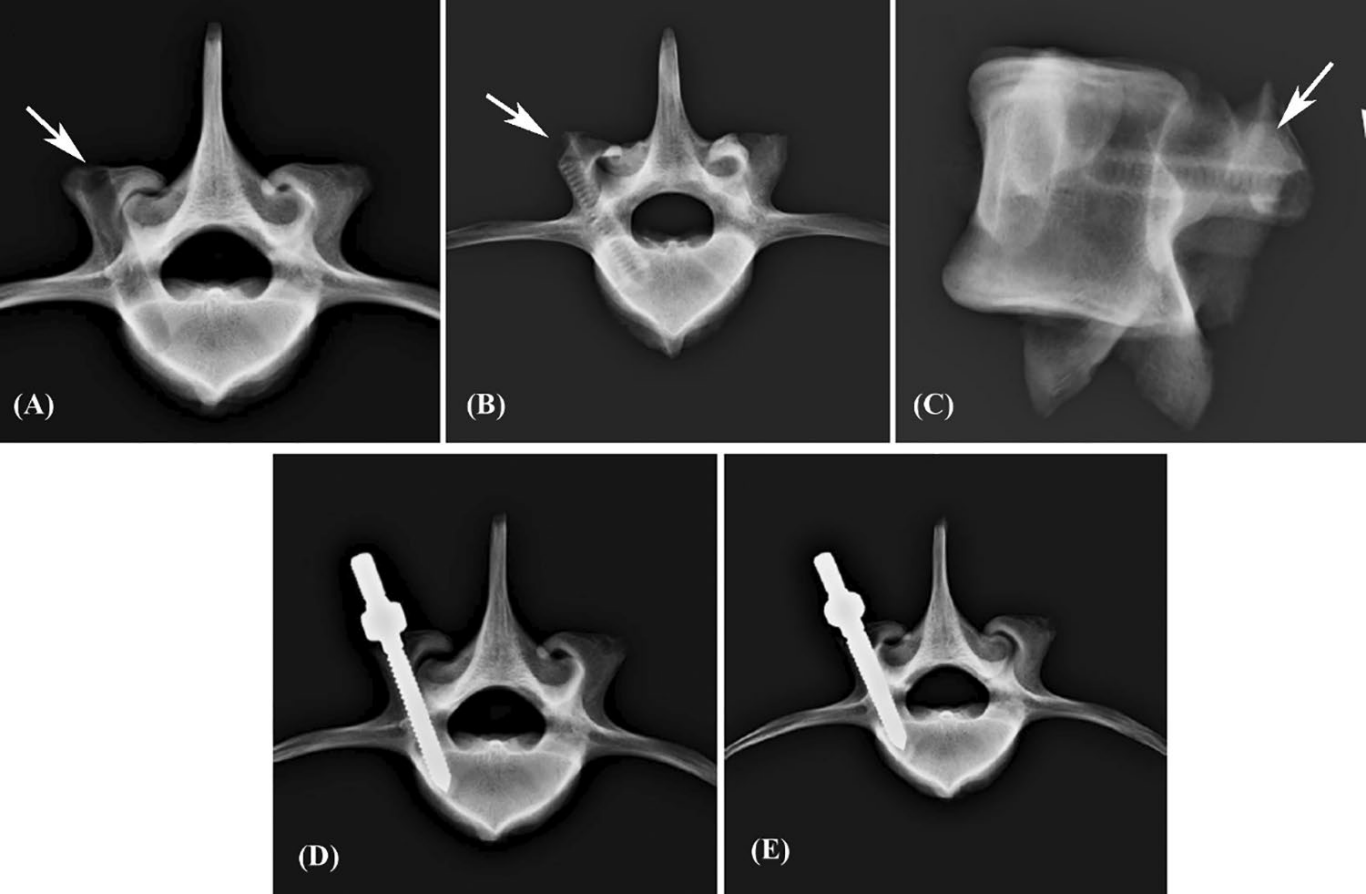
X-ray images of the modified group. An enlarged screw hole was prepared and confirmed using axial X-rays (**A**) (white arrow). A stiff threaded PMMA hole was confirmed by axial (**B**) and sagittal (**C**) X-ray images (white arrow). A cylindrical (**D**) or conical (**E**) repair screw was convergently implanted. The tip of the inserted screw did not exceed the enlarged depth, and no signs of fracture or damage were detected in the vertebrae.

An appropriate screw trajectory and insertional depth were confirmed using axial and sagittal X-ray imaging prior to pullout testing (Figs. 1, 2). In the traditional group, a 5.5 mm diameter enlarged screw hole was prepared (Fig. 1A, B), and cylindrical (Fig. 1C) and conical (Fig. 1D) repair screws were convergently inserted into the vertebral body along the track. The tip of the inserted screw did not exceed the enlarged depth, and no signs of fracture or damage were detected in the vertebrae.

In the modified group, a 5.5 mm diameter enlarged screw hole was formed (Fig. 2A), and a threaded PMMA screw hole was created by tapping screws (Fig. 2B, C). The two thread-type repair screws were inserted into the vertebral body individually in each group, and no fractures or defects were detected in either view (Fig. 2D, E).

### Pullout strength

**Figure 3:**
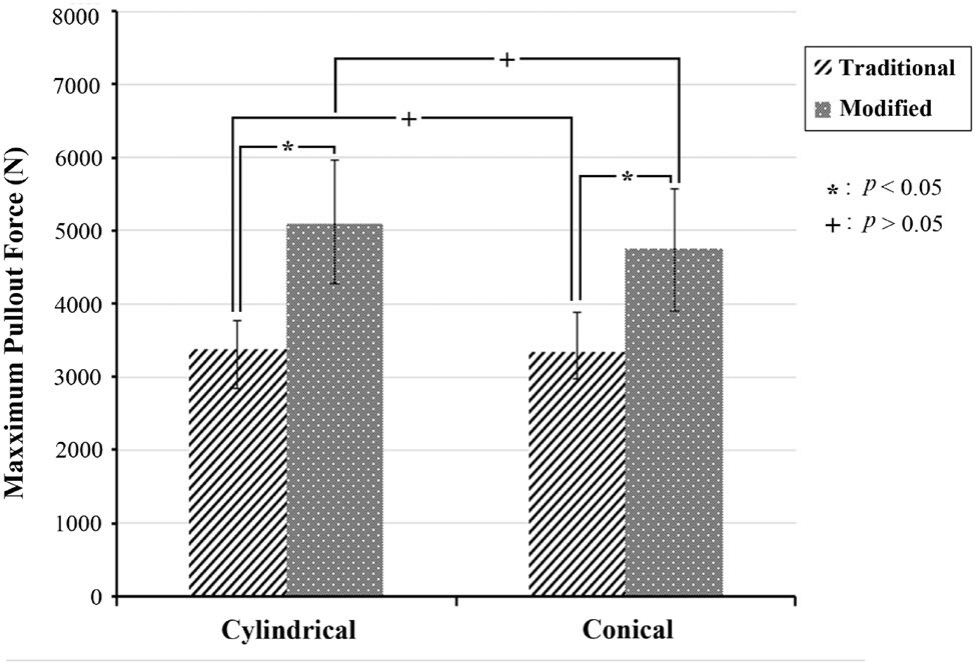
Mean maximum pullout forces for cylindrical and conical screws in the traditional and modified groups. In the traditional group, the cylindrical and conical repair screws had mean pullout strength values of 3386.90 ± 370.94 N and 3338.80 ± 548.37 N, respectively; these values were 5095.63 ± 859.67 N and 4755.99 ± 819.60 N in the modified group, respectively. (Groups without statistically significant difference are indicated with “+” symbol).

**Figure 4:**
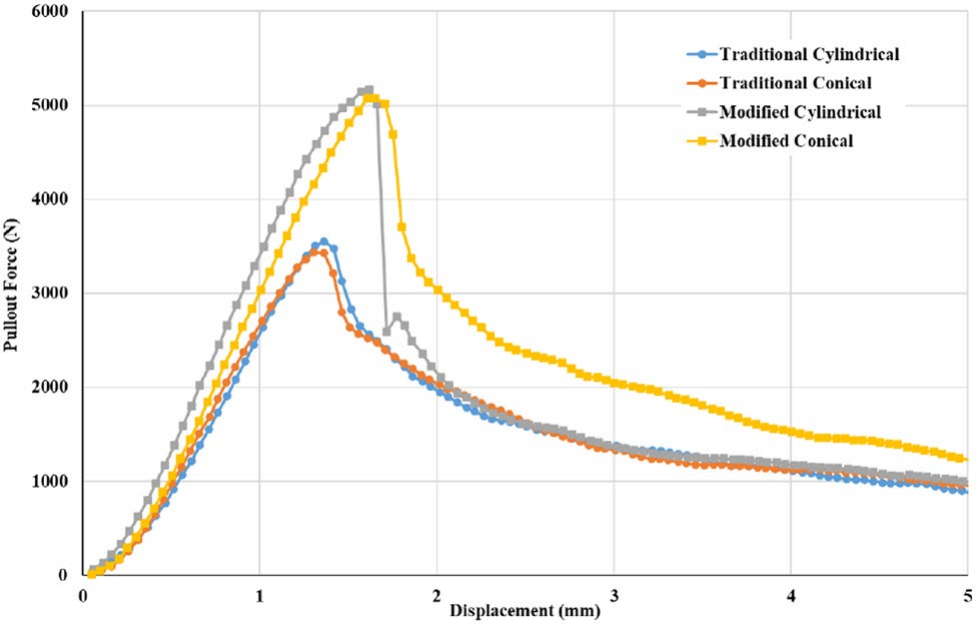
Typical force–displacement curve for cylindrical and conical screws in the traditional and modified groups. For a given screw geometry, the pullout force for the modified group was significantly higher than that for the traditional group; however, there were no statistically significant differences for the two types of screws in either the traditional or the modified filling method.

In the traditional group, cylindrical and conical repair screws had mean pullout strength values of 3386.90 ± 370.94 N and 3338.80 ± 548.37 N, respectively; these values were 5095.63 ± 859.67 N and 4755.99 ± 819.60 N in the modified group, respectively (Fig. 3). The maximal pullout force for the modified group was 150% for the cylindrical screw and 142% for the conical screw relative to those for the traditional group. For a given screw geometry, the pullout force for the modified group was significantly higher than that for the traditional group; however, there was no statistically significant difference in pullout force between the screw geometries for a given filling technique. The typical force–displacement curves for cylindrical and conical screws in the traditional and modified groups are shown in Fig. 4. For a given screw geometry, the pullout force for the modified group was significantly higher than that for the traditional group; however, there was no statistically significant difference in pullout force between the screw geometries for a given filling technique.

## Discussion

Screw loosening is one of the most frequently reported complications of pedicle screw fixation. Several surgical strategies can be performed to treat the effects of loosening screws, i.e., implant failure and subsequent clinical symptoms^16,17^.

Replacement of loosened screws with thicker, longer screws is commonly used but still limited by insecure fixation of the largest available screws^3^. According to biomechanical and clinical studies, use of PMMA cement is an effective procedure for the repair of pedicle screws in bone. Empty cancellous bone can be replaced by PMMA and reach immediate postoperative stability. However, devastating clinical symptoms such as pulmonary embolism and neurological deficits can occur when PMMA cement is improperly or excessively injected and leaks along broken pedicle holes^5,18–20^. Fewer cement-related complications and immediate stability of repairs are major goals for the repair of loose pedicle screws using PMMA augmentation.

**Figure 5:**
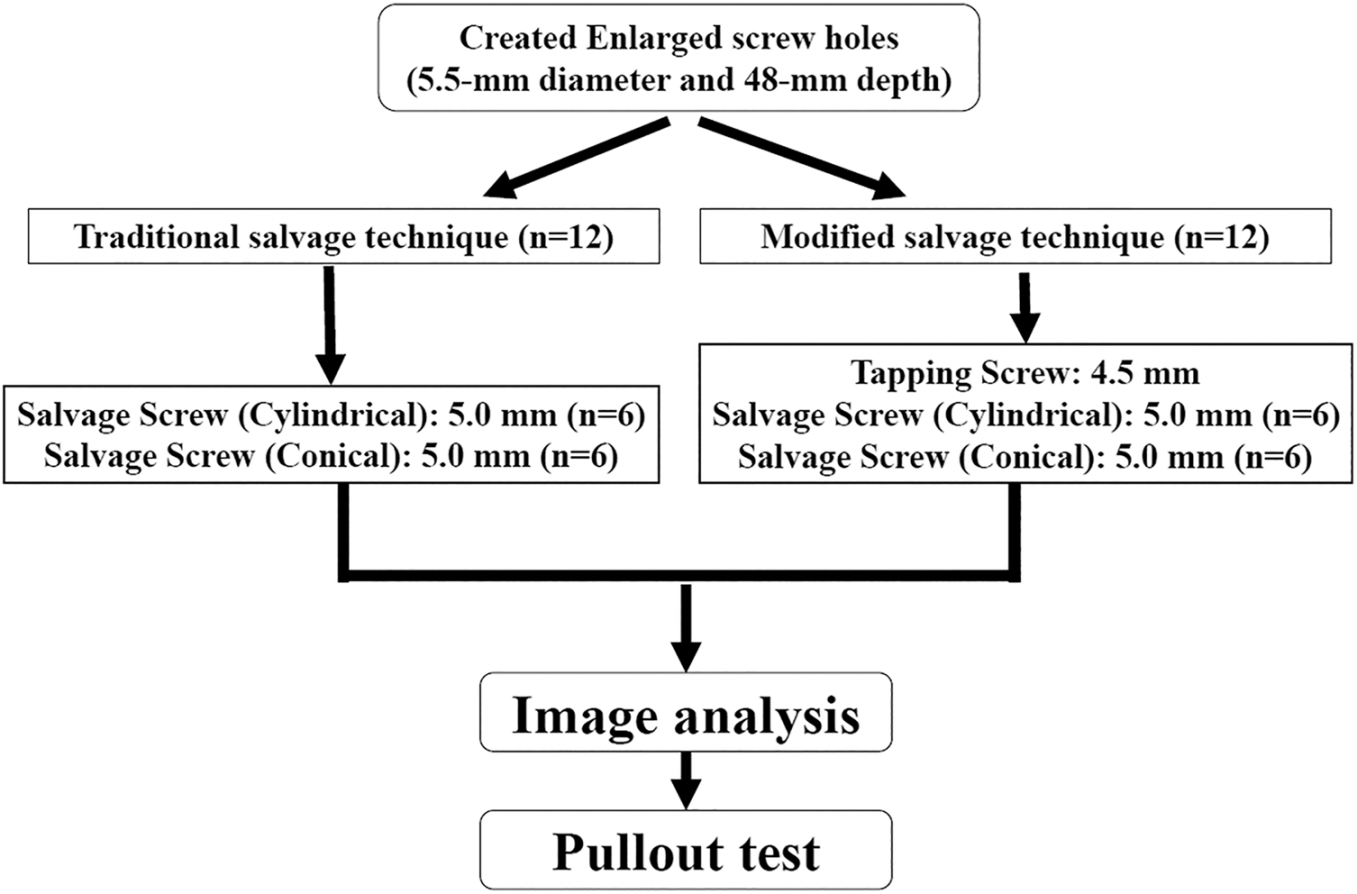
A flowchart of the study design.

In our study, a randomized trial of sufficient size using porcine vertebrae with adequate amounts of cement filling proved that there was a statistically significant improvement in stability in the modified group over the traditional group. The porcine spine is a useful biomechanical model of the human spine because of the similar geometric characteristics of the vertebral body, spinal canal, and pedicle shape^21^. In this study, we used a universal self-aligning fixture (Fig. 5) to achieve coaxial alignment of the pedicle screw with the pullout ram. We ensured that the screws and the direction of pullout force were along the same axis prevent an 8% error by mounting onto a material testing machine at different angles^22^. The imaging results showed that all the repair screws in both groups were inserted along the loosening track without penetrating the cancellous bone, and there were no signs of fracture or damage; therefore the subsequent application of the pullout force would reflex only the force of bone-cement or screw-cement interface without interference.

The traditional cement filling method has been clinically applied as a repair for loosening screws and demonstrated to lead to a 0.5–2.0-fold increase in pullout strength biomechanically^23,24^. In our study, the pullout force of the modified group was considerably higher than that of the traditional group by 1.4–1.5-fold, which means that the method can be applied clinically.

Several repair methods using PMMA have been conducted and compared to increase the additional anchor pullout force for pedicle screw loosening^5,14,25^. Fenestrated PMMA-augmented screws have been compared with traditional solid screws, but the results were inconsistent due to the variation in the sizes, numbers and location of hole and uneven cement infiltration^5,14^. Increased epidural leakage of cement with fenestrated screws in cadaveric spines also limited further clinical applications^22^. Vikas T et al. reported novel PMMA-augmented solid screws with a bicortical fixation method; in their study, 364 screws were used in 40 patients, and no complications were recorded^25^. However, theoretical risks including vascular injury associated with anterior cortical penetration, are still a crucial concern. PMMA-augmented expansive screws have been reported to have a better pullout strength than the traditional PMMA filling method in the calve spine, but uncontrolled screw loosening tracks and unequal screw size resulted in unreliable results^26^.

In our study, implantation of a repair screw after the PMMA setting time was crucial for fixation stability. Failed PMMA augmentation was reported to result from bone-cement interface failure due to uncured PMMA cement or the screw-cement interface failure related to cured cement^27,28^. The increased anchoring effect of the screw-cement interface from the tapping screw to the repair screw were proved by the better higher pullout performance in our modified group.

Previous studies have reported that the outer shape of screws has an effect on the pullout strength, leading to the conclusion that a conical thread compressed the surrounding bone during screw insertion, thereby increasing pullout strength, which was further confirmed with finite element analyses^29,30^. In our study, the distal part of the repair screw was larger than the tapping screw, and more pullout strength was supposed to be produced. However, for a given filling technique, there was no notable difference in pullout force between screw geometries. Our results indicate that the cement augmentation technique was far more influential than the screw outer geometry, which was consistent with the results of other studies^14,31^.

The present study has some limitations. First, the pullout force in porcine vertebrae cannot totally represent that of humans. In actual physiological situations, the vertebrae are subjected to complex multi-directional loading. Further investigation of the effects of different loading conditions, such as cyclic multi-directional loading, would be beneficial for long-term evaluation. Second, our data represent only biomechanical testing with normal bone density; clinically common osteoporotic bone density was not examined. Third, to preserve the uniformity and reproducibility of the screw loosening pattern, a 5.5 mm diameter straight screw hole was formed, which might not represent the true screw loosening pattern with an uneven radiolucent surrounding area. Fourth, more diameter differences should be examined to evaluate the effect of cement. Fifth, the PMMA filling pattern or interdigitation into cancellous bone could not be fully controlled in either group. Lastly, only one volume (0.8 ml) of PMMA cement was examined; further investigations with various cement volumes and larger sample sizes might be necessary.

## Conclusions

Our study shows the pullout strength was higher in the modified group than in the traditional group, and the cement augmentation was far more influential than the screw outer geometry. We believe that this modified filling technique could be an important alternative for revision of pedicle screw loosening.

## Materials and methods

### Specimen preparation

This study was performed using twenty-four single-level fresh frozen lumbar vertebrae from L1 to L6 that were harvested from four mature pigs (weight 100–120 kg). All animals were healthy before harvesting and never exposed to any drugs or procedures that could affect bone density. All the specimens were separated into individual vertebrae after stripping away the surrounding musculature, ligaments, and periosteum. All the specimens were embedded in 10% formalin solution (Sigma-HT501640, Sigma Chemical Co.) for 24 h before the biomechanical and imaging studies. An enlarged screw hole was prepared in one side of each vertebra, which was drilled using a 5.5 mm “twist” metric drill bit attached to a Dremel 4000 rotary tool mounted on a Dremel WorkStation Model 220–01. This trajectory was selected based on previously reported morphometric characteristic data^32^. The track was followed with a standard straight pedicle probe to a depth of 48 mm.

### Specimen grouping and screw insertion

**Figure 6:**
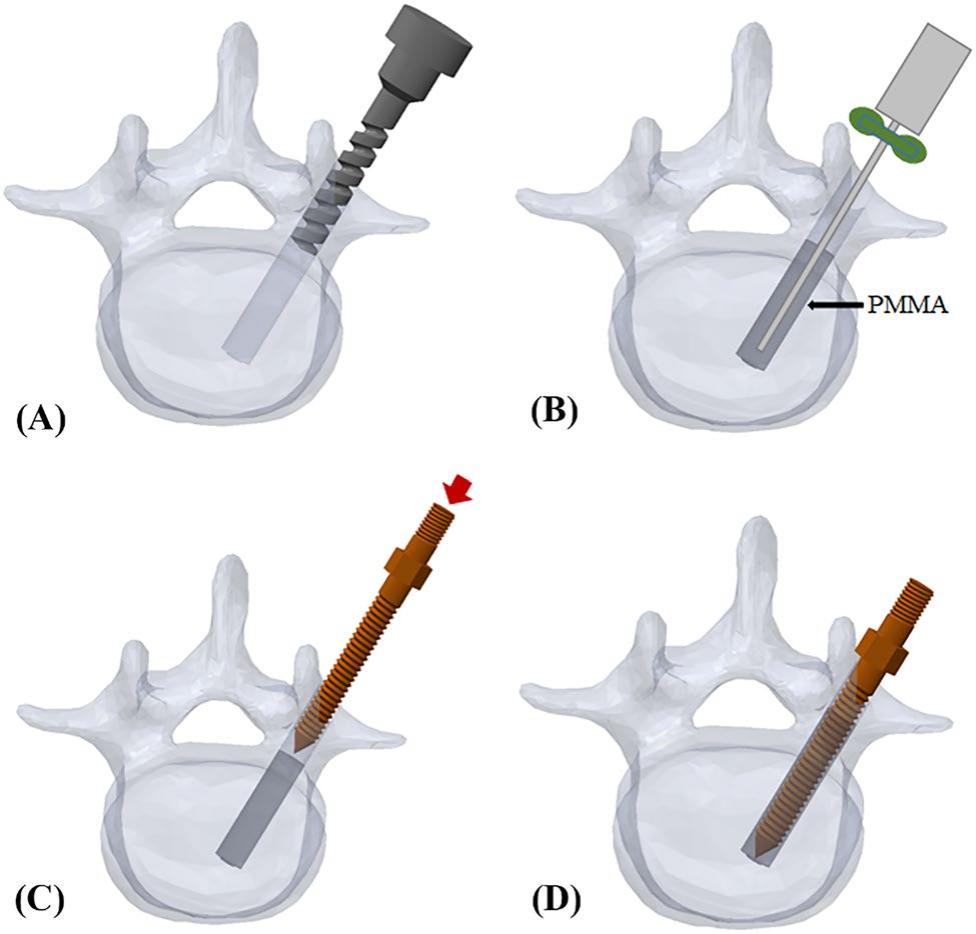
Schematic illustrations of the traditional group. The 48 mm depth enlarged screw tract was created by 5.5 mm twist metric drill bit (**A**). A total of 0.8 ml of PMMA (with consistency similar to that of toothpaste) was retrograde injected into the enlarged screw hole (**B**). A diameter × length of 5.0 mm × 45 mm salvage screw was gradually implanted into the PMMA-filled screw hole (**C**) until all threads were fully covered (**D**).

**Figure 7:**
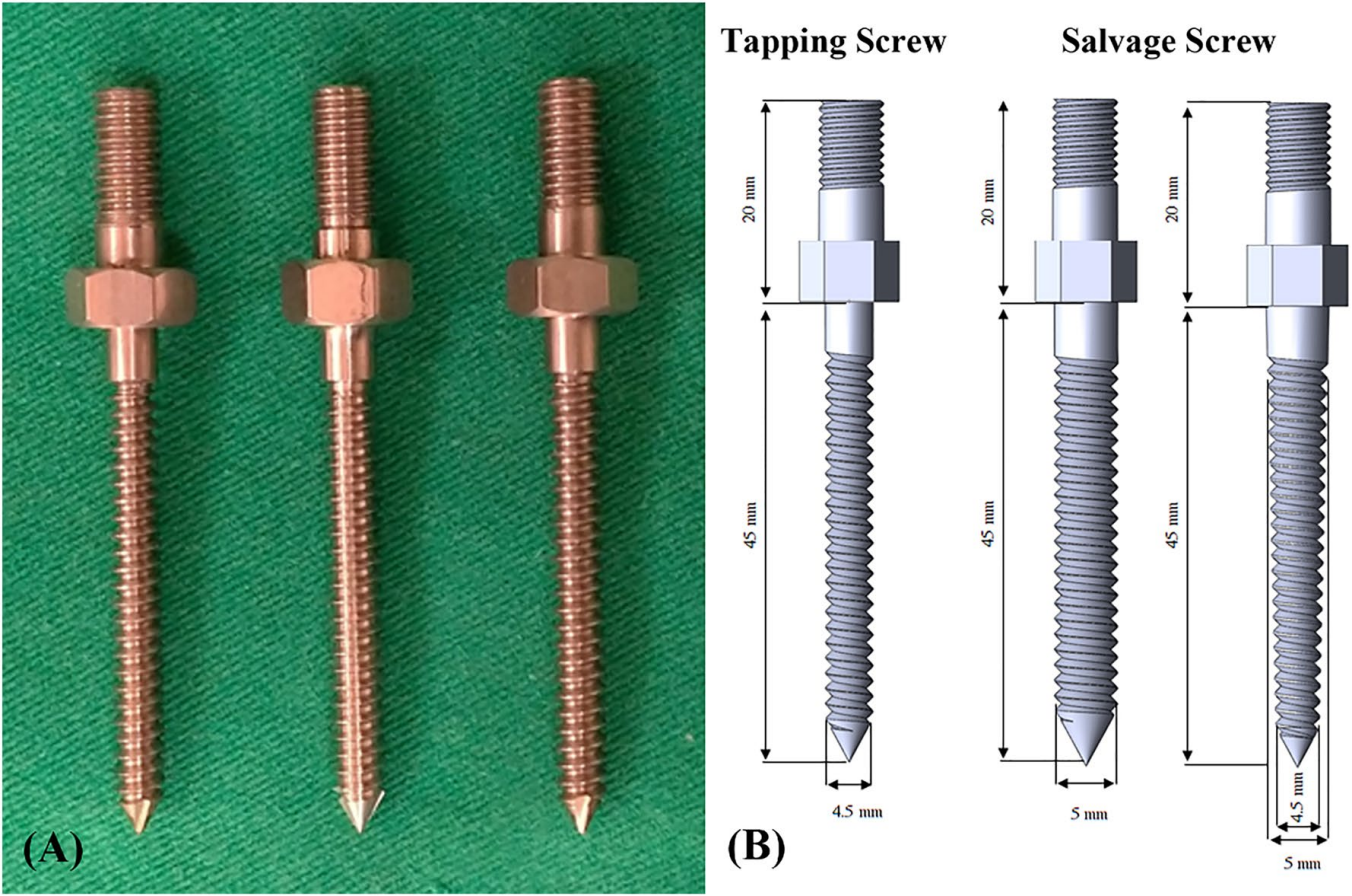
Salvage screws and tapping screw. Two salvage screw designs were employed in the present study: cylindrical and conical shaped (**A** and **B**, middle and right). The outer geometry of the conical and cylindrical screws differed mainly in the taper of their major and minor diameters from the hub to the screw tip. The cylindrical screws maintained a constant diameter from hub to tip (**A** and **B**, middle); in contrast, the conical screws tapered 10%, from 5.0 mm at the hub to 4.5 mm at the tip (**A** and **B**, right). The tapping screw was cylindrical and maintained a diameter of 4.5 mm from hub to tip (**A** and **B**, left). For all screw designs, the thread pitch was 2 mm and the thread depth was 0.8 mm.

**Figure 8:**
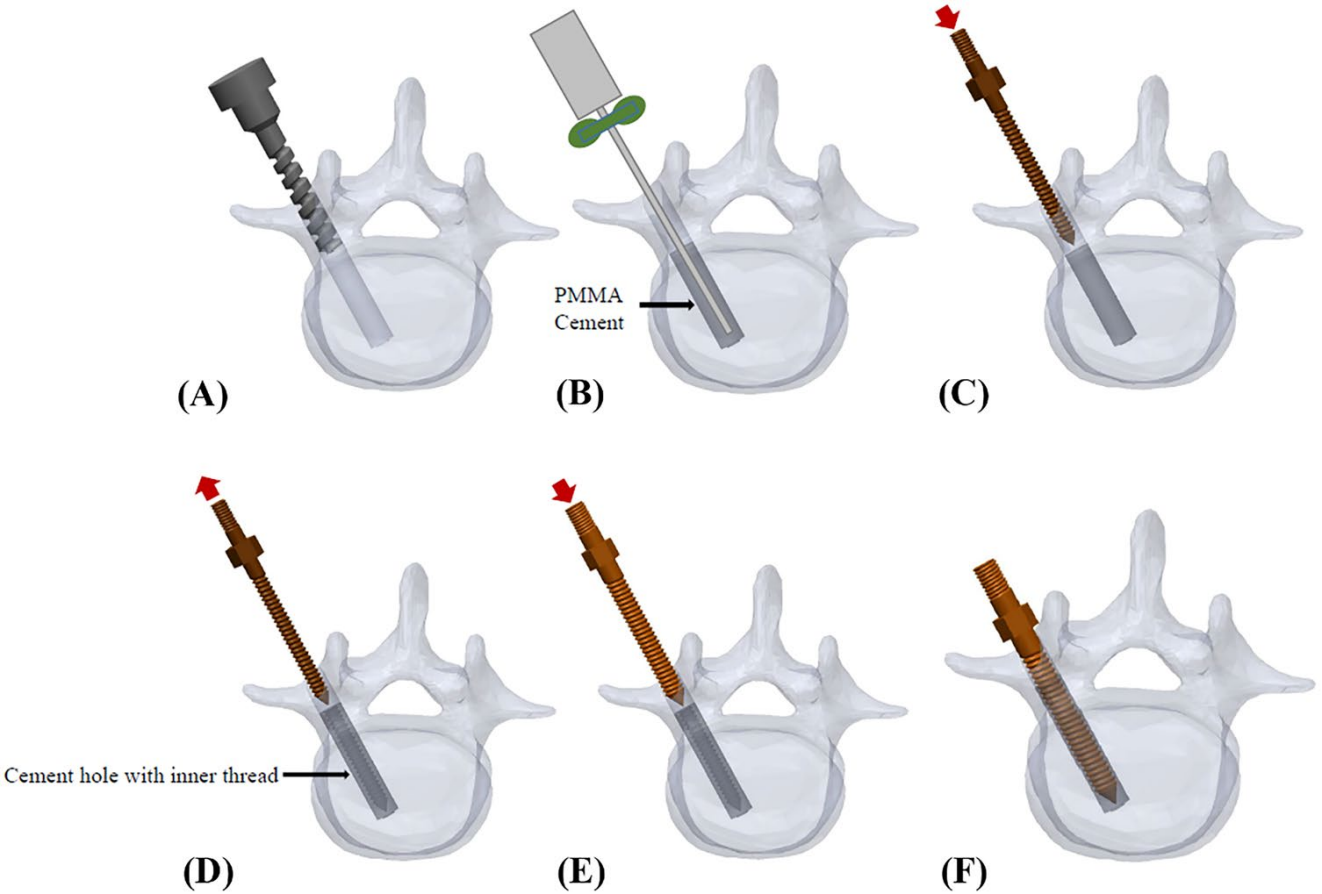
Schematic illustrations of the modified group. The 48 mm deep enlarged screw tract was formed by a 5.5 mm twist metric drill bit (**A**), and 0.8 ml PMMA was retrograde injected into the enlarged screw hole (**B**). A tapping screw was inserted into the PMMA- filled hole (**C**). After setting for 7 min, the screw was carefully removed, leaving a stiff threaded PMMA hole (**D**). A cylindrical or conical repair screw, sized according to that used in the traditional technique, was implanted (**D**, **E**). Vertebra with complete insertion of the repair screw (**F**).

Twelve specimens were randomly allocated to two cement filling techniques: the traditional technique or the modified technique. Cylindrical or conical screws with identical thread types were used to evaluate the effect of screw geometries in 6 specimens of each group (Fig. 5). In the traditional group, 0.8 ml PMMA was retrograde injected into the enlarged screw holes (Fig. 6). A cylindrical or conical salvage screw with a diameter × length of 5.0 mm × 45 mm (SmartLoc spinal polyaxial pedicle screws, A-spine Asia Co. Ltd., Taipei, Taiwan) (Fig. 7) was chosen and randomly implanted into each pedicle of the vertebrae by an experienced surgeon. In the modified group, a tapping screw with a diameter × length of 4.5 mm × 45 mm was inserted after injection of 0.8 ml PMMA into the enlarged screw hole. After the 7-min setting time of the PMMA^33^, the screw was carefully removed, leaving a stiff threaded PMMA hole. A cylindrical or conical repair screw sized according to that used in the traditional technique was implanted (Fig. 8). Before the pullout test, the specimens and screws were closely examined for signs of fracture and damage, and any findings were carefully recorded.

### Image analysis

Axial and sagittal views were examined via X-rays for all specimens prior to the pullout test to confirm an appropriate screw trajectory and insertion depth. The vertebrae were also examined thoroughly to exclude any fractures or defects caused by screw insertion.

### Pullout test

**Figure 9:**
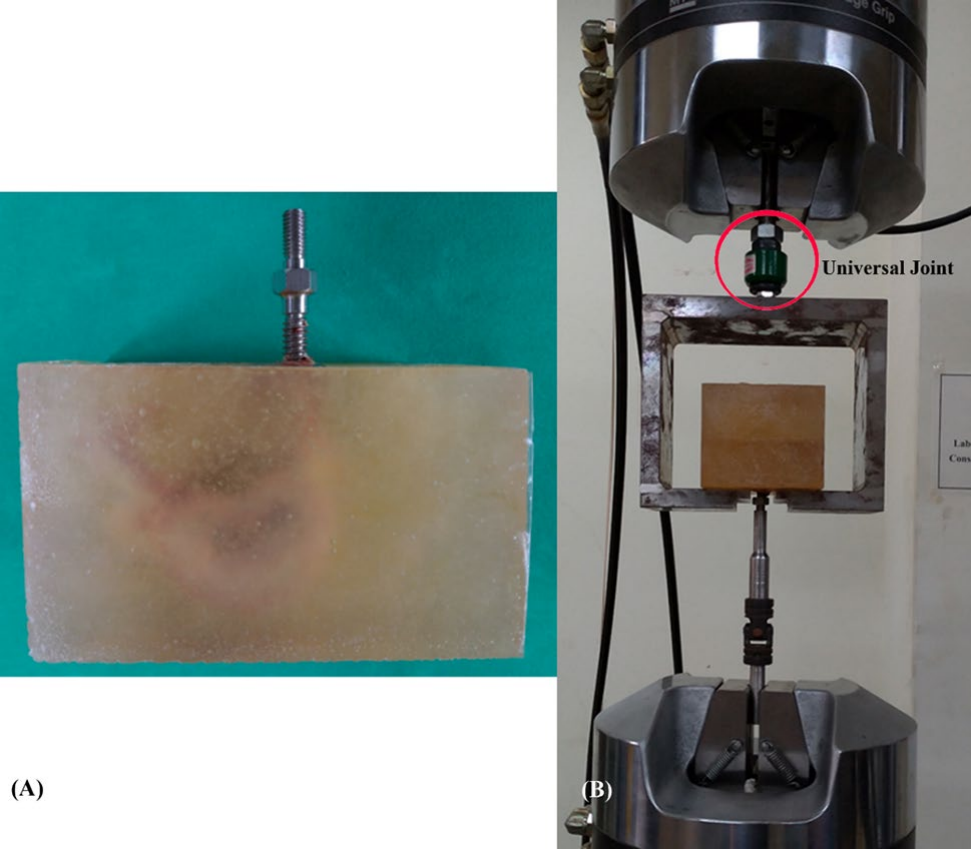
Photographs of a cubic specimen embedded in acrylic mixture (**A**), and the screw pullout test performed by MTS testing machine (**B**). The custom-designed universal joint with a self-aligning function to ensure vertical pullout measurement is highlighted.

Each of the 24 specimens was potted in a metal box using a specific acrylic mixture (Buehler, Lake Bluff, IL, USA) to prepare a cubic specimen with dimension of 8 cm × 8 cm × 6 cm (Fig. 9A). Judicious potting was performed to ensure that the acrylic mixture did not contact any portion of the screw, and the specimens were potted into acrylic acid with a cubic shape to fit in the square clamp below the universal joint. The prepared specimens were mounted onto a material testing machine (Bionix 858; MTS Systems Corp., MN, USA) to conduct axial pullout tests with the screws (Fig. 9B). The method for the screw pullout test was identical to that used in our previous study^14^. The embedded specimen, with a screw inserted, was placed on a custom-designed universal fixture with a self-aligning function to ensure vertical pullout alignment. The head of the screw was attached to the testing machine by a threaded rod assembly. After the specimens were mounted, the pullout force was applied at a constant crosshead rate of 5 mm/min for a total displacement of 10 mm, which was in accordance with published literature on axial pull out testing^34,35^. Data collection was set at 1 sample/0.05 mm (1.67 Hz). Failure was defined as the maximum load or the load peak prior to a decrease in load associated with increasing displacement^36^. After the pullout test was completed, the specimen and screws were closely examined for signs of fracture, and any damage was carefully recorded.

### Statistical analysis

Statistical software (SPSS for Windows version 12.0, SPSS Inc., Chicago, IL) was used to analyze the pullout force of all the specimens. All of the measurements were collected for 24 vertebrae and the results were expressed as the mean ± standard deviation (SD). An ANOVA test with post hoc analyses was performed to evaluate the differences between groups. Differences were considered statistically significant at *p* < 0.05.
